# Supplementary material for: Dynamic models augmented by hierarchical data: an application of estimating HIV epidemics at sub-national level
Source: Biostatistics. 2024 Feb 29;25(4):1049–61. doi: 10.1093/biostatistics/kxae003 (PMC11471966; doi:10.1093/biostatistics/kxae003)
Supplement: kxae003_Supplementary_Data [file kxae003_supplementary_data.pdf]

Biostatistics (2023), **0**, 0, *pp.* 1–**??**
doi:10.1093/biostatistics/output

Appendix for "Dynamic Models Augmented by Hierarchical Data: An Application Of Estimating HIV Epidemics At Sub-National And Sub-Population Level"

LE BAO* and Xiaoyue Niu

Department of Statistics, Penn State University, University Park, PA, USA

Tim Brown

Population and Health Studies, East-West Center, Honolulu, HI, USA

Jeffrey W. Eaton

MRC Centre for Global Infectious Disease Analysis, School of Public Health, Imperial College London, London, UK

lebao@psu.edu

APPENDIX

A. Full Data Analysis Results For Nigeria

In Nigeria, we model the HIV prevalence among pregnant women in 37 areas, present the full data analysis result for one area in the main manuscript and present the results for the rest of the areas here. Some fitted curves do not go through the observed antenatal clinic data because they are pulled away by the HIV prevalence estimated in the national survey.

*To whom correspondence should be addressed.

© The Author 2023. Published by Oxford University Press. All rights reserved. For permissions, please e-mail: journals.permissions@oup.com

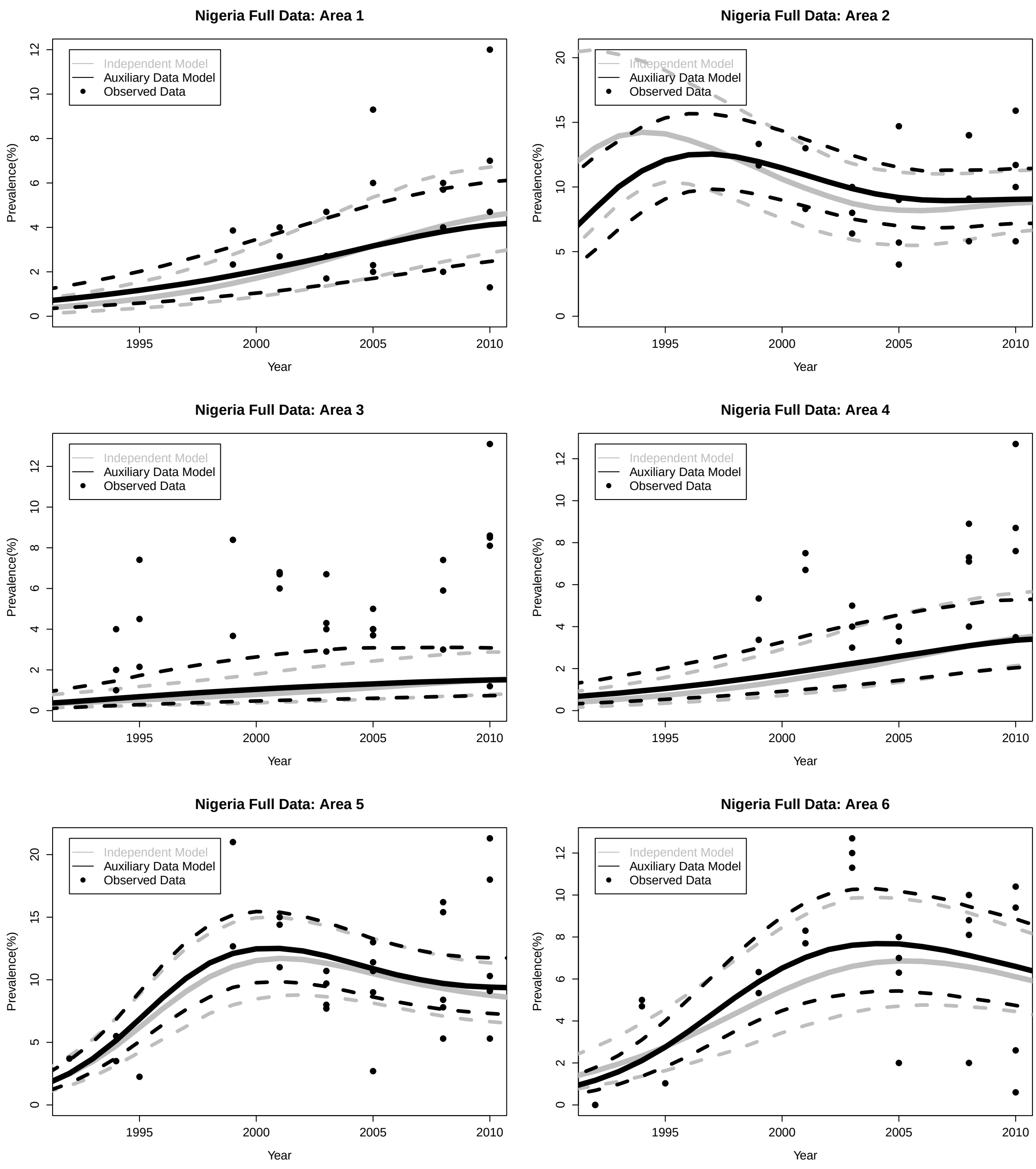

Figure 1. Full Data Analysis Results For Nigeria. The gray curves show the posterior median and 95% credible interval of prevalence trends estimated from the original EPP model without using auxiliary data; the black curves show the posterior median and 95% credible interval of prevalence trends estimated from EPP augmented by auxiliary data; the black dots show the observed data.

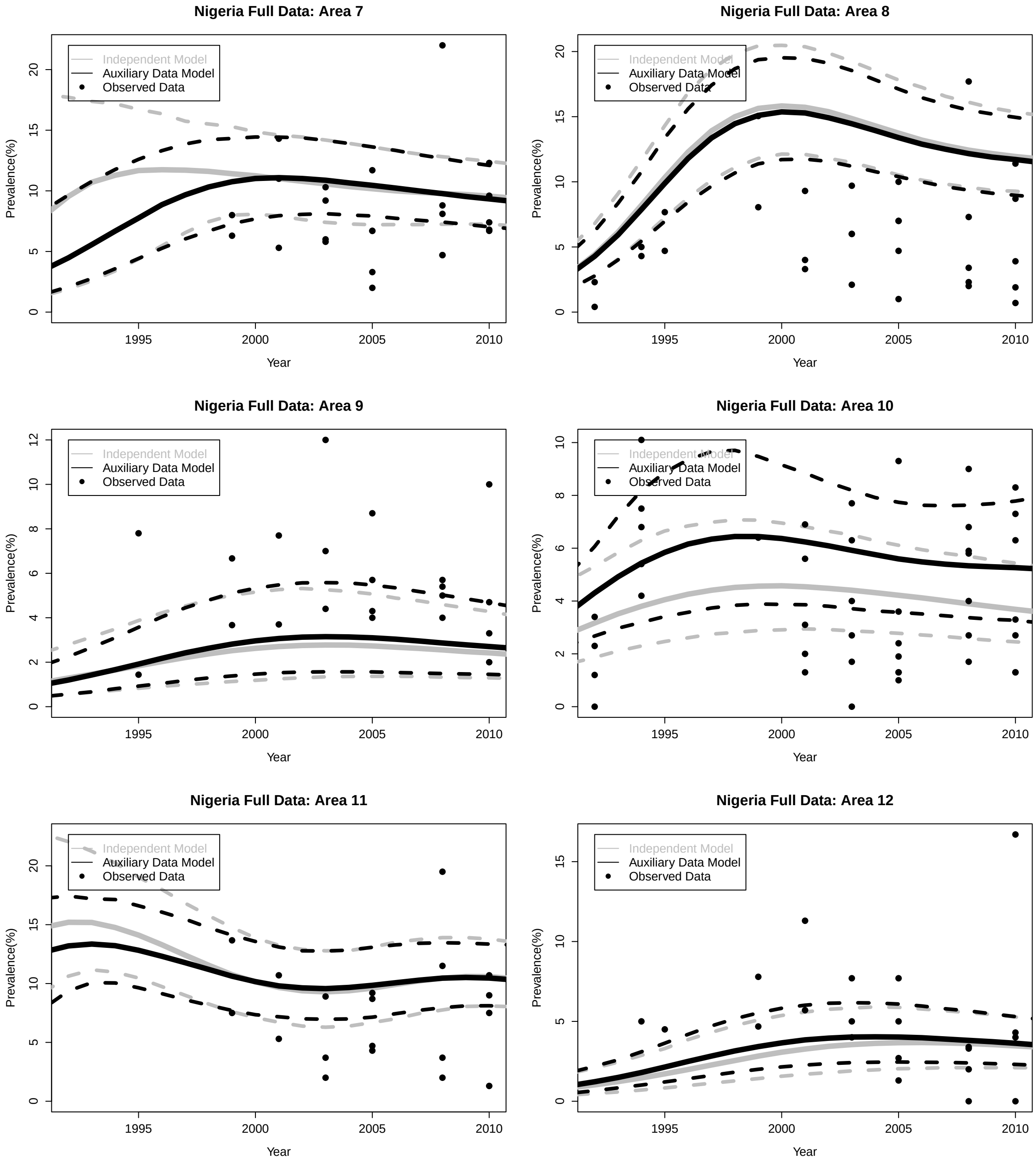

Figure 2. Full Data Analysis Results For Nigeria. The gray curves show the posterior median and 95% credible interval of prevalence trends estimated from the original EPP model without using auxiliary data; the black curves show the posterior median and 95% credible interval of prevalence trends estimated from EPP augmented by auxiliary data; the black dots show the observed data.

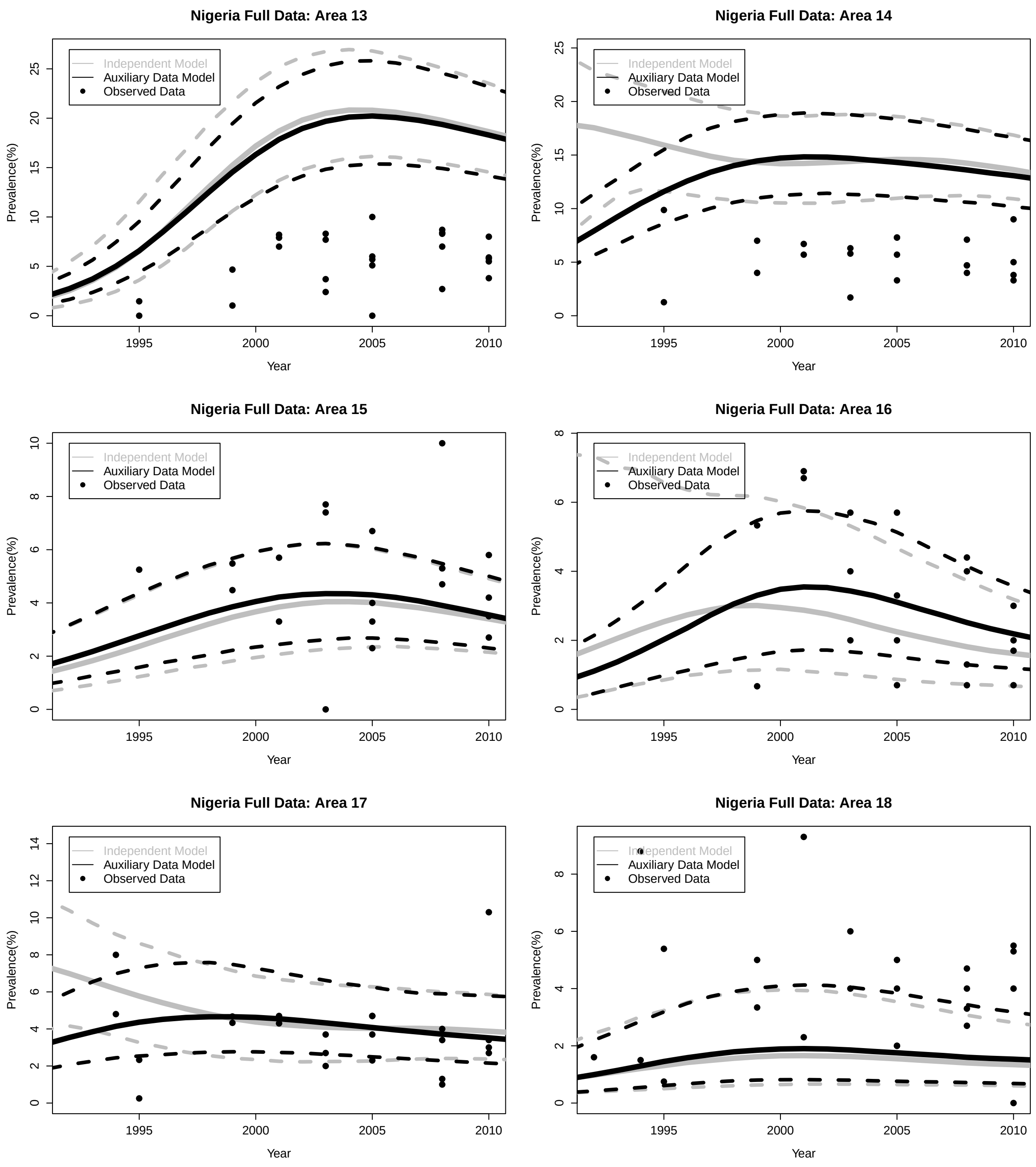

Figure 3. Full Data Analysis Results For Nigeria. The gray curves show the posterior median and 95% credible interval of prevalence trends estimated from the original EPP model without using auxiliary data; the black curves show the posterior median and 95% credible interval of prevalence trends estimated from EPP augmented by auxiliary data; the black dots show the observed data.

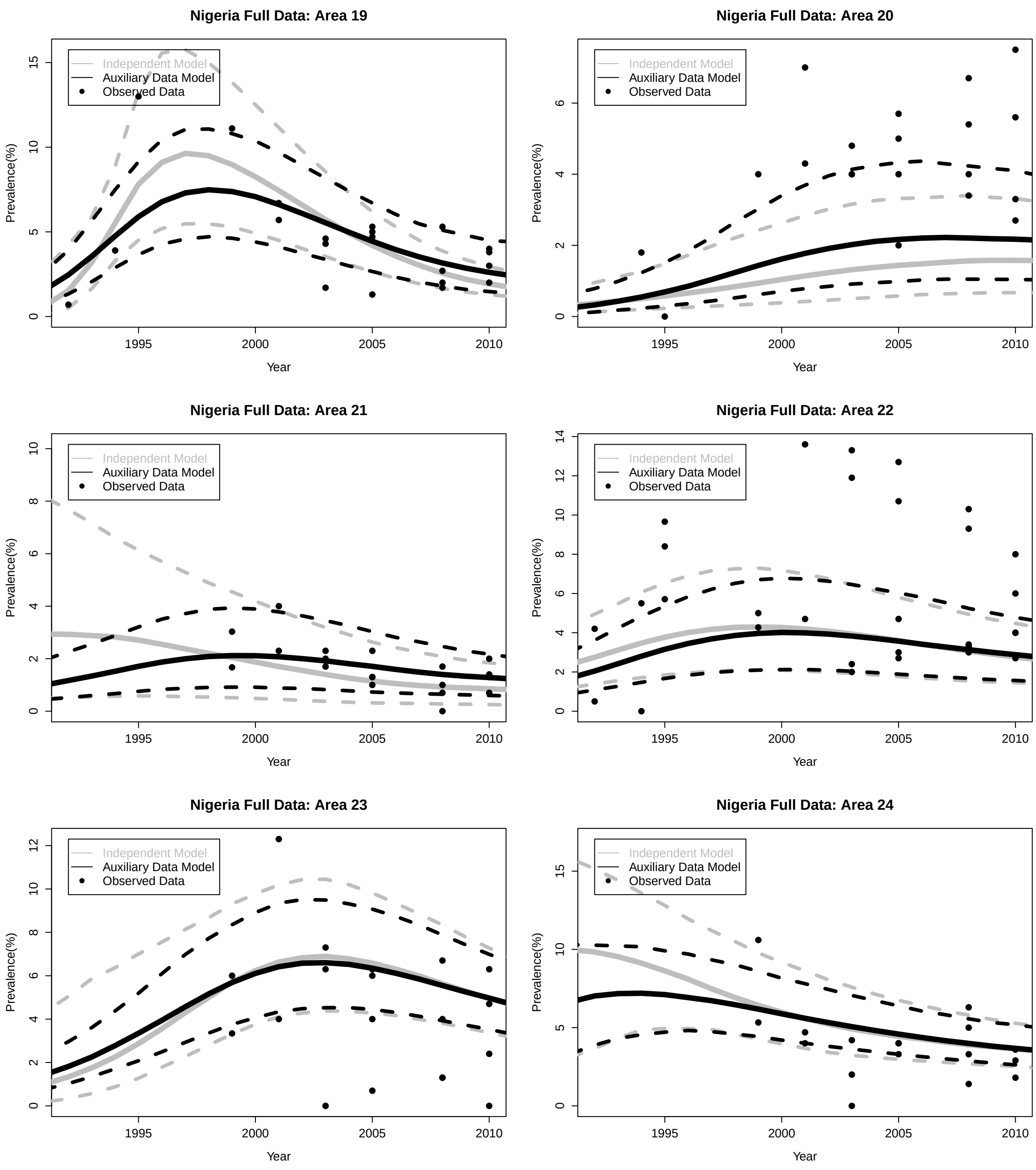

Figure 4. Full Data Analysis Results For Nigeria. The gray curves show the posterior median and 95% credible interval of prevalence trends estimated from the original EPP model without using auxiliary data; the black curves show the posterior median and 95% credible interval of prevalence trends estimated from EPP augmented by auxiliary data; the black dots show the observed data.

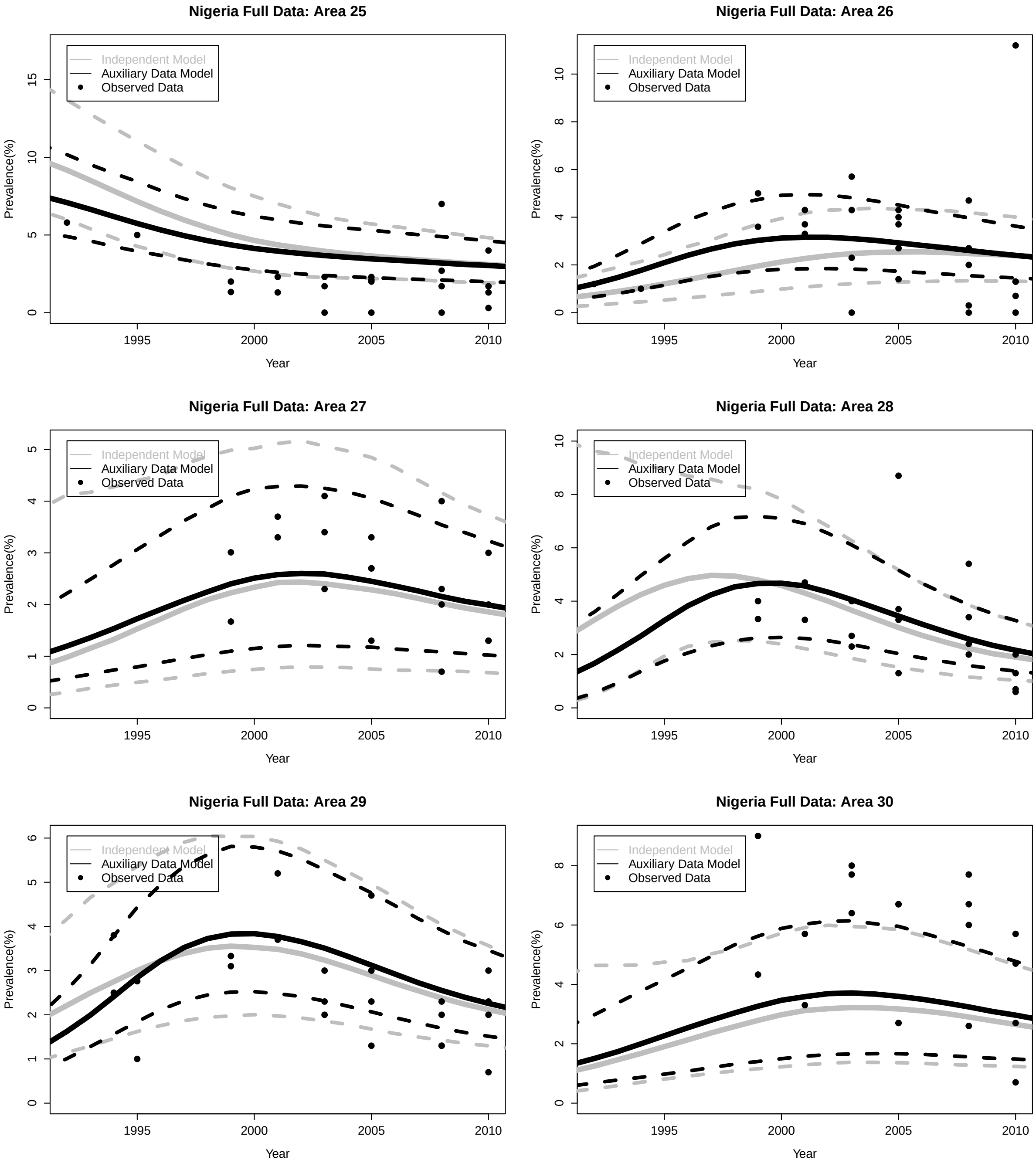

Figure 5. Full Data Analysis Results For Nigeria. The gray curves show the posterior median and 95% credible interval of prevalence trends estimated from the original EPP model without using auxiliary data; the black curves show the posterior median and 95% credible interval of prevalence trends estimated from EPP augmented by auxiliary data; the black dots show the observed data.

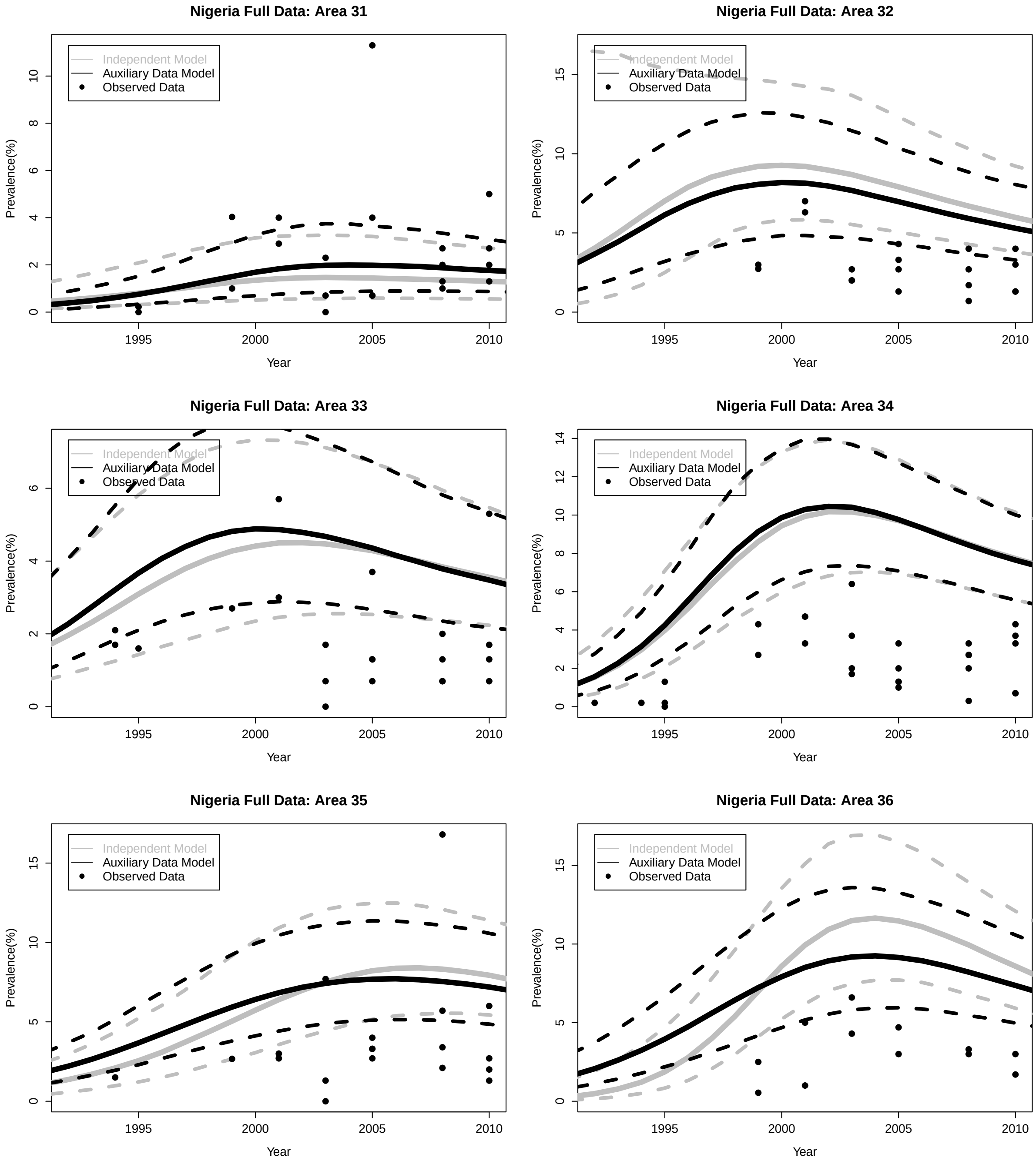

Figure 6. Full Data Analysis Results For Nigeria. The gray curves show the posterior median and 95% credible interval of prevalence trends estimated from the original EPP model without using auxiliary data; the black curves show the posterior median and 95% credible interval of prevalence trends estimated from EPP augmented by auxiliary data; the black dots show the observed data.
